# Investigating the Neurotoxic Impacts of Arsenic and the Neuroprotective Effects of Dictyophora Polysaccharide Using SWATH-MS-Based Proteomics

**DOI:** 10.3390/molecules27051495

**Published:** 2022-02-23

**Authors:** Jun Zhang, Ting Hu, Yi Wang, Xinglai Zhang, Huajie Zhang, Jing Lin, Xiaoxiao Tang, Xukun Liu, Margy Chen, Naseer Ullah Khan, Liming Shen, Peng Luo

**Affiliations:** 1 The Key Laboratory of Environmental Pollution Monitoring and Disease Control, School of Public Health, Ministry of Education, Guizhou Medical University, Guiyang 550025, China; zj805549879@163.com (J.Z.); huting@gmc.edu.cn (T.H.); 18198363621@163.com (Y.W.); zxl18886072401@163.com (X.Z.); 2 College of Life Science and Oceanography, Shenzhen University, Shenzhen 518060, China; zhj9609@126.com (H.Z.); 1900251011@email.szu.edu.cn (J.L.); 2060251043@email.szu.edu.cn (X.T.); lxk06@szu.edu.cn (X.L.); arieschemist@yahoo.com (N.U.K.); 3 Department of Psychology, Emory University, Atlanta, GA 30322, USA; margy.chen@emory.edu

**Keywords:** SWATH, NaAsO_2_, dictyophora, polysaccharides, neurotoxicity

## Abstract

Arsenic (As) is one of the most important toxic elements in the natural environment. Currently, although the assessment of the potential health risks of chronic arsenic poisoning has received great attention, the research on the effects of arsenic on the brain is still limited. It has been reported that dictyophora polysaccharide (DIP), a common bioactive natural compound found in dietary plants, could reduce arsenic toxicity. Following behavioral research, comparative proteomics was performed to explore the molecular mechanism of arsenic toxicity to the hippocampi of SD (Sprague Dawley) rats and the protective effect of DIP. The results showed that exposure to arsenic impaired the spatial learning and memory ability of SD rats, while DIP treatment improved both the arsenic-exposed rats. Proteomic analysis showed that arsenic exposure dysregulated the expression of energy metabolism, apoptosis, synapse, neuron, and mitochondria related proteins in the hippocampi of arsenic-exposed rats. However, DIP treatment reversed or restored the expression levels of these proteins, thereby improving the spatial learning and memory ability of arsenic-exposed rats. This study is the first to use high-throughput proteomics to reveal the mechanism of arsenic neurotoxicity in rats as well as the protective mechanism of DIP against arsenic neurotoxicity.

## 1. Introduction

Arsenic (As) is a common environmental toxicant and occupational contaminant that affects over 200 million people worldwide, primarily in developing countries, and arsenic poisoning is still a world public health issue [1]. The accumulation of arsenic in the human body can cause organ damage and tissue carcinogenesis. While it enhances the production of reactive oxygen species (ROS) such as superoxide and hydrogen peroxide, it increases protein oxidation, disrupts enzymes and DNA [2], causes lipid peroxidation, impairs mitosis, and promotes apoptosis.

Arsenic can easily cross the blood–brain barrier and accumulate in various parts of the brain including the striatum and hippocampus, amplifying arsenic toxicity and tissue injury [3]. Long-standing evidence has shown the role of hippocampal neurons in the formation of spatial learning and memory. By impairing hippocampal neurons, arsenic toxicity leads to decreased learning and memory capacity [4]. Arsenic, when present in high concentrations in the brain, disrupts neurocellular signaling pathways and functions, resulting in induced synaptic transmission and neurological dysfunction [5]. However, there is still no consensus on the exact mechanism of arsenic-induced neurotoxicity.

Chronic arsenic poisoning is caused by long-term consumption of contaminated food and water, and there is currently no effective treatment for symptoms. Recent research has found that bioactive natural compounds found in dietary plants can improve arsenic-induced toxicity [6], and their medicinal value is gaining in popularity. Among them, dictyophora polysaccharide (DIP) was found to have anti-inflammatory, anti-tumor, and immunomodulatory activities [7,8,9]. Recent studies have noted that it has a neuroprotective effect [10,11]. However, the effect of DIP on the neurotoxicity caused by sodium arsenite is still unknown.

In this study, the learning and memory capability of arsenic exposed-SD (Sprague Dawley) rats was shown to be impaired, but DIP alleviated the impairment. To investigate the molecular mechanism of arsenic-induced hippocampus toxicity and the protective effect of DIP on arsenic-induced neurotoxicity, a comparative proteomics analysis based on SWATH (sequential windowed acquisition of all theoretical fragment ion mass spectra) was performed. SWATH-MS (mass spectrum) analysis was chosen because of its high reproducibility and quantitative accuracy.

## 2. Results

### 2.1. DIP Improved Spatial Learning and Memory of Arsenic Treated Rats

**Figure 1:**
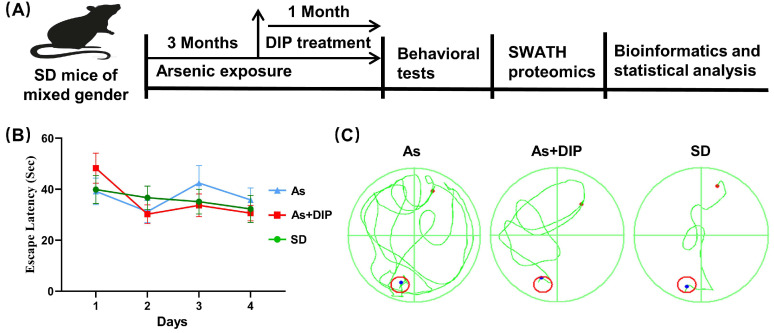

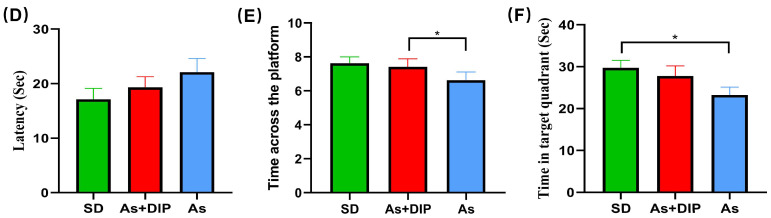
Schematics for study design and behavioral tests. (**A**) Schematic diagram of the whole experiment. (**B**) The escaping latencies of rats in the 4-day trials. (**C**) The representative swimming track lines of rats in the 4-day trials. (**D**) The escaping latencies of rats. (**E**) The times of rats crossing over the hidden location of the platform. (**F**) The time of mice spent in the target quadrants. * *p* < 0.05, n = 20. (**D**–**F**) In the probe trail at 24 h after the 4-day successive training.

In the Morris Water Maze (MWM) test, compared to the control group, the swimming track lines of the arsenic-treated group were more irregular with much higher escape latency (Figure 1B,C). The latency on the fifth day was also higher in the arsenic-treated group (Figure 1D). Following that, a probe trial was conducted to assess the rats’ memory 24 h after the 4-day successive training. The DIP + As group rats crossed the platform’s hidden location much more frequently than the arsenic-treated group rats (Figure 1E). In addition, the DIP + As group rats spent significantly more time in the target quadrants than the arsenic-treated rats (Figure 1F).

### 2.2. Overview of Protein Expression Characteristics of Different Groups

**Figure 2:**
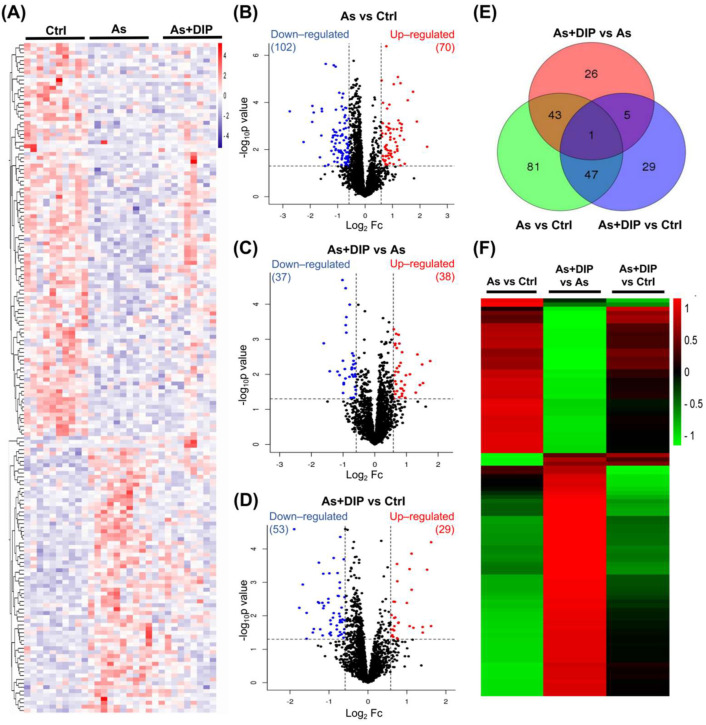
Differentially expressed proteins identified in different groups. (**A**) Cluster map comparing the proteins identified in the As/Ctrl, DIP + As/As, and DIP + As/Ctrl groups. Red color indicates higher expression (upregulation), blue indicates lower expression (downregulation), and white indicates similar expression levels. (**B**) Volcano plots depicted the distribution of proteins in the As/Ctrl group. (**C**) Volcano plots depicted the distribution of proteins in the As + DIP/As group. (**D**) Volcano plots depicted the distribution of proteins in the As + DIP/Ctrl group. For parts (**B**–**D**), the log_2_ fold change (FC) is plotted versus the −log_10_ of the *p*-value. Red dots = hits with *p* < 0.05 and mean log_2_ FC > 0.58; blue dots = hits with *p* < 0.05 and mean |log_2_ FC| < 0.58. (**E**) Venn diagrams of DEPs among the As/Ctrl group, As + DIP/As group, and As + DIP/Ctrl. (**F**) The cluster analysis results of different groups of DEPs.

SWATH-MS analysis identified a total of 3833 quantitative proteins with 2674 quantitative proteins meeting the quality control requirements after QC correction. We then compared and analyzed the protein expression profiles between different groups including the arsenic-treated group versus the control group (As/Ctrl group); DIP treatment + NaAsO_2_-treated group compared with the NaAsO_2_-treated group (DIP + As/As group); and DIP treatment + NaAsO_2_-treated group compared with control group (DIP + As/Ctrl group). The cluster analysis revealed that the protein expression pattern of the As/Ctrl group differed significantly from that of the other two groups, whereas the DIP + As/As group and the DIP + As/Ctrl group exhibited more similar patterns (Figure 2A).

Among them, 172, 75, and 82 proteins were identified as DEPs in the As/Ctrl group, DIP + As/As group, and DIP + As/Ctrl groups, respectively (Figure 2B–D). Of these, 44 DEPs were found to be common between the As/Ctrl group and DIP + As/As group, while 43 DEPs were discovered to have the opposite expression trend in these two groups (named as reversed proteins; Table 1, Figure 2E). When compared to the As/Ctrl group, the expression of most DEPs in the As/Ctrl group was restored or reversed in the As + DIP/As group (Table 1, Figure 2F).

### 2.3. Differentially Expressed Proteins between NaAsO_2_-Treated Group and Control Group (As/Ctrl Group)

**Figure 3:**
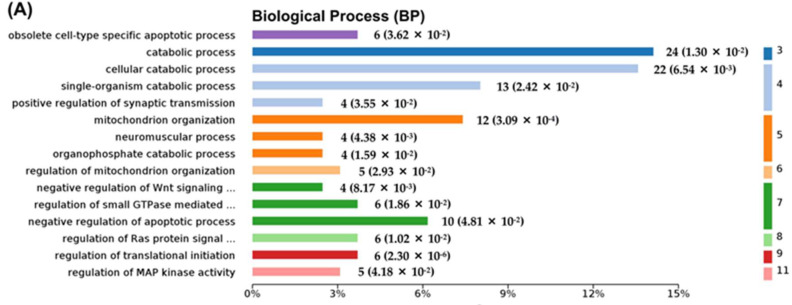

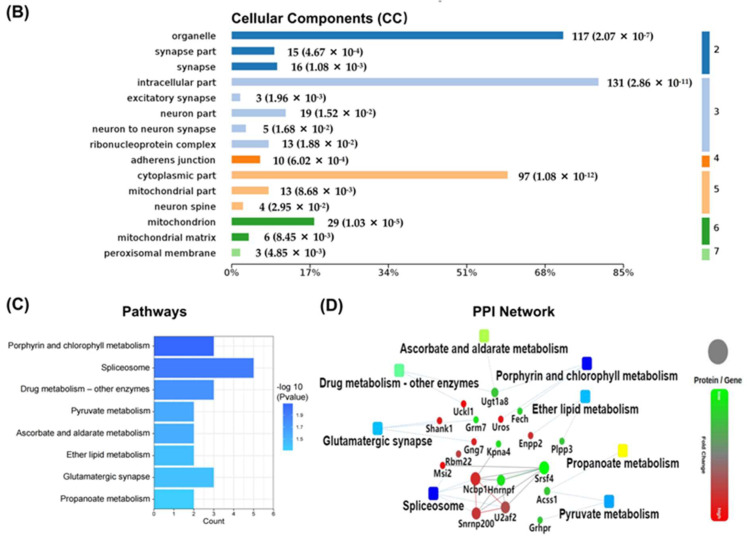
Bioinformatics analysis of the differentially expressed proteins in the As/Ctrl group. (**A**) The top 15 categories of enriched BP associated with DEPs. (**B**) The top 15 categories of enriched CC associated with DEPs. (**C**) The significantly enriched KEGG pathways linked to DEPs. (**D**) Pathway PPI network linked to the differentially expressed proteins in the As/Ctrl group. (**A**–**C**) The number of proteins and *p* values associated with each category is shown to the right of each term bar.

According to the bioinformatics analysis, the biological process (BP) associated with DEPs in the As/Ctrl group was mainly involved in the cellular catabolic process and regulation of apoptotic process, regulation of translational, protein and enzyme signal regulation, and organophosphate catabolic process. Several signal transduction pathways were involved including Ras protein signal transduction, Wnt signaling pathway, small GTPase mediated signal transduction, and MAP kinase activity (Figure 3A, Table S1). Among the DEPs, 12 were related to mitochondrion organization including Ddhd2, Pmpca, Bcs1l, Ndufaf7, Slc25a46, Hars2, Elmod1, Tfam, U2af2, Bag3, Fam162a, and Msto1; 10 DEPs (i.e., Lrp1, Krt18, Grm7, Itgb1, Eif2ak2, Bag3, Rps6ka1, Map4k4, Anp32b, and Cav1) were related to negative regulation of apoptotic process; four DEPs (i.e., Unc13a, Shank1, Tbc1d24, and Clstn1) were associated with the regulation of synaptic transmission; and two DEPs (including Kcna1 and Fgf12) were involved in the neuronal action potential process (Table S1). By cellular components (CC) analysis, the DEPs were mainly distributed in the cytoplasm, cytoplasmic parts, mitochondrion, and mitochondrial matrix. Noticeably, multiple DEPs were distributed in synapse and neuron part: 16 DEPs belonged to synaptic proteins and 10 of them were downregulated; 19 DEPs belonged to the neuronal part and 14 of them were downregulated (Figure 3B, Table S2). These DEPs were found to be involved in metabolic pathways and glutamatergic synapse. The metabolic pathways included pyruvate metabolism, propanoate metabolism, ether lipid metabolism, ascorbate, aldarate metabolism, and drug metabolism-other enzymes (Figure 3C). Ten DEPs were found to be involved in these metabolic pathways with six of them being downregulated (Table S3). Figure 3D depicts the protein−protein interaction (PPI) network related to DEPs. The findings agreed with the Kyoto Encyclopedia of Genes and Genomes (KEGG) pathway analysis. 

**Figure 4:**
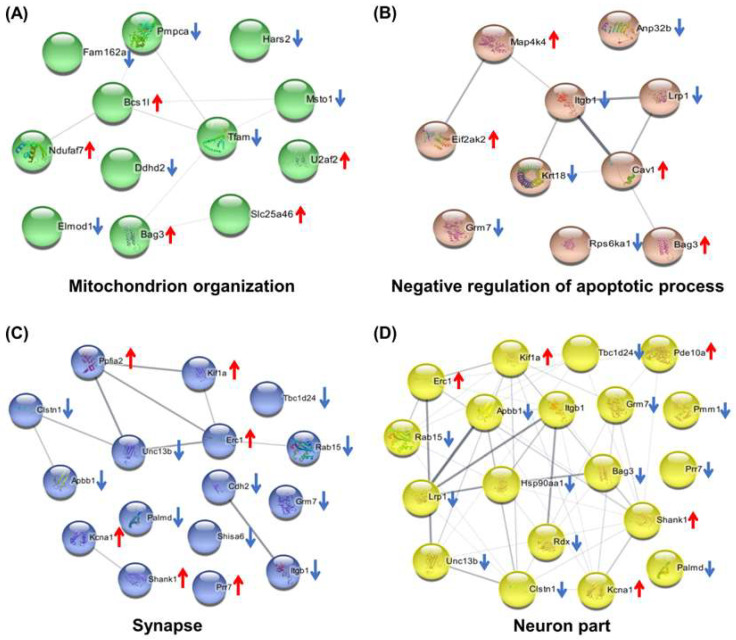
Bioinformatics analysis of the differentially expressed proteins in the As/Ctrl group. (**A**–**D**) PPI of DEPs involved in mitochondrion organization, negative regulation of apoptotic process, synapse, and neuron part, respectively. ↑, upregulated; ↓, downregulated.

We further analyzed the DEPs enriched into the key pathway of Gene Ontology (GO) analysis. The PPI of DEPs involved in mitochondrion organization, negative regulation of apoptotic process, and synapse and neuron parts are shown in Figure 4A–D, respectively.

### 2.4. Differentially Expressed Proteins between DIP + As-Treated Group and As-Treated Group (DIP + As/As Group)

**Figure 5:**
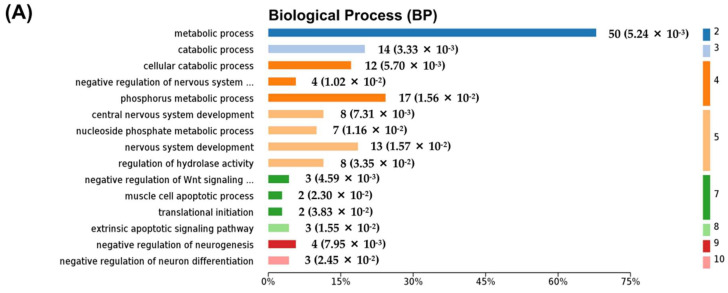

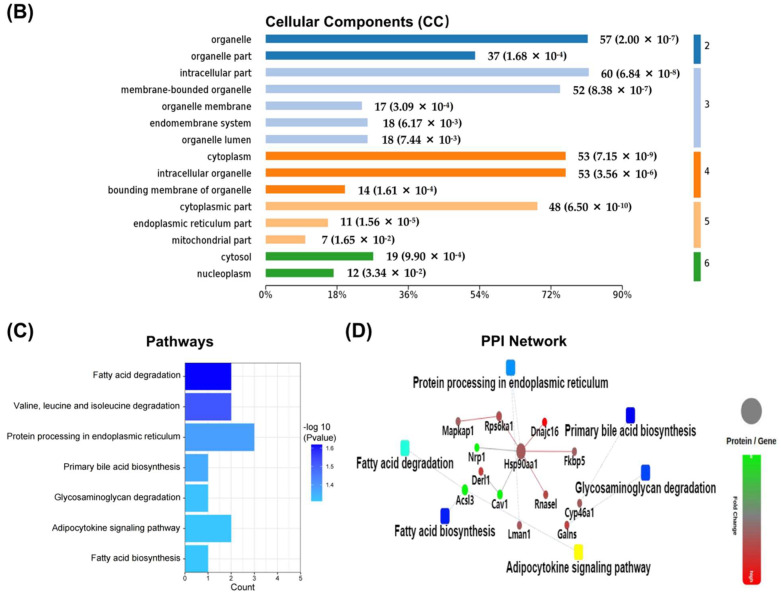
Bioinformatics analysis of the differentially expressed proteins in the As + DIP/As group. (**A**) The top 15 categories of enriched BP associated with DEPs. (**B**) The top 15 categories of enriched CC associated with DEPs. (**C**) The significantly enriched KEGG pathways linked to DEPs. (**D**) Pathway PPI network linked to the differentially expressed proteins in the As + DIP/As group. (**A**–**C**) The number of proteins and *p*-values is shown to the right of each term bar.

**Figure 6:**
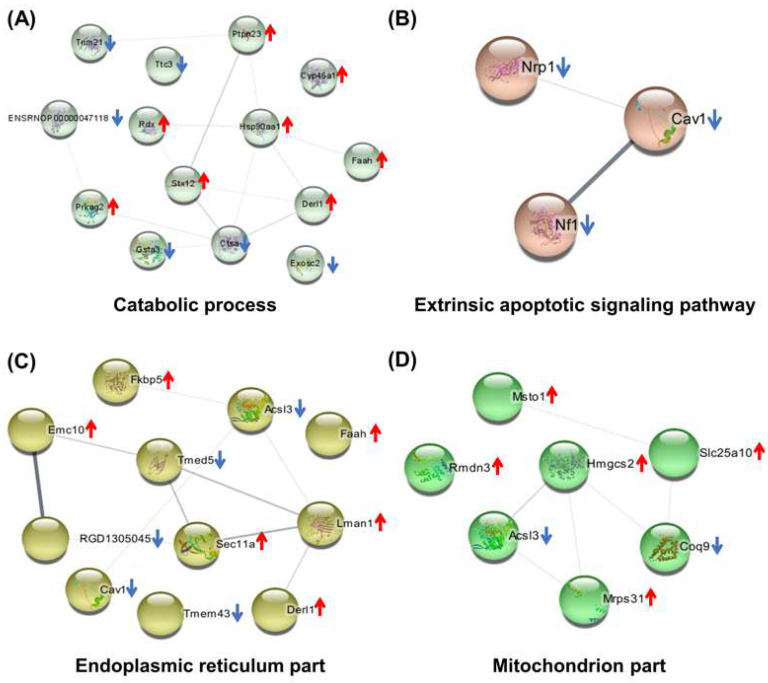
Bioinformatics analysis of the differentially expressed proteins in the As + DIP/As group. (**A**–**D**) PPI of DEPs involved in the catabolic process, extrinsic apoptotic signaling pathway, and endoplasmic reticulum and mitochondrion parts, respectively. ↑, upregulated; ↓, downregulated.

The DIP + As/As group contained 75 DEPs. The BP associated with these DEPs was mainly involved in the metabolic process, catabolic process, regulation of hydrolase activity, translational initiation, extrinsic apoptotic, Wnt signaling pathway, nervous system development, and regulation of neuron differentiation. These were widely distributed in cells. Among them, 50 were involved in the metabolic process (Figure 5A, Table S4), seven were mitochondrial part proteins, and 11 were endoplasmic reticulum part proteins (Figure 5B, Table S5). According to the KEGG and PPI network, they were linked to glycosaminoglycan degradation pathways, protein processing in the endoplasmic reticulum, and fatty acid biosynthesis and degradation (Figure 5C,D, Table S6). The PPI of DEPs involved in catabolic process, the extrinsic apoptotic signaling pathway, and the endoplasmic reticulum and mitochondrion parts are shown in Figure 6A–D, respectively.

### 2.5. Differentially Expressed Proteins between DIP + As-Treated Group and Control Group (DIP + As/Ctrl Group)

We also compared the protein expression profiles of the DIP + As-treated group and the control group. Eighty-two DEPs were identified. Compared with the results from the As/Ctrl comparison, about half of the DEPs suggested that DIP treatment changed the protein expression profile in the hippocampi of rats exposed to arsenic, turning it closer to normal expression.

### 2.6. The Reversed Proteins between the As/Ctrl Group and DIP + As/As Group

**Figure 7:**
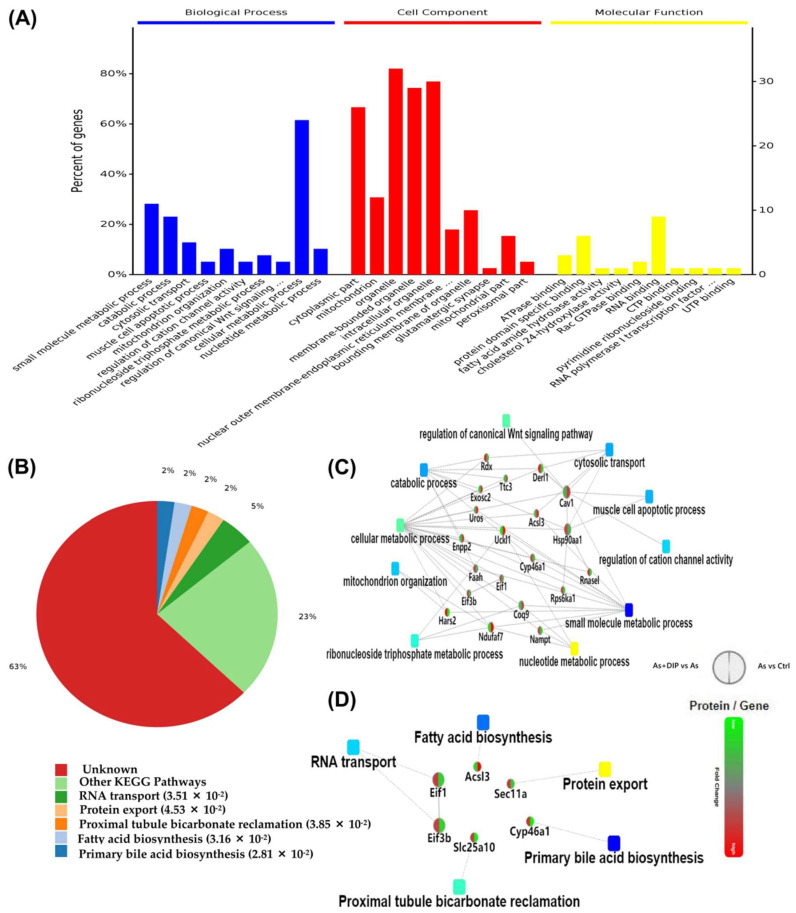
Bioinformatics analysis of the reversed proteins between the As/Ctrl, and DIP + As/As group. (**A**) The GO annotations of the reversed proteins. (**B**) The KEGG pathways associated with the reversed proteins. (**C**) Biological process PPI network linked to the reversed proteins in this group. (**D**) Pathway PPI network linked to the reversed proteins in this group.

Subsequently, we analyzed the 43 reversed proteins between the As/Ctrl group and DIP + As/As group. Among these, 22 proteins were upregulated and 21 proteins were downregulated in the As/Ctrl group, while their expression trends were reversed in the DIP + As/As group. GO analysis showed that these proteins were mainly implicated in the catabolic process and apoptotic process, and mitochondrion organization (Figure 7A, Table S7), and were widely distributed in cells. They were involved in the regulation of biosynthesis pathways and other biological processes such as RNA transport and protein export (Figure 7B, and Table S8). The BP-related PPI network revealed that these reversed proteins were associated with the catabolic process, mitochondrion organization, apoptotic process, and other signaling pathways. According to the PPI network and KEGG analysis, these proteins were correlated with protein export, acid biosynthesis, and RNA transport pathways (Figure 7C,D).

### 2.7. Hub Genes Analysis and Cluster Analysis of Differentially Expressed Proteins in Key Pathways

**Figure 8:**
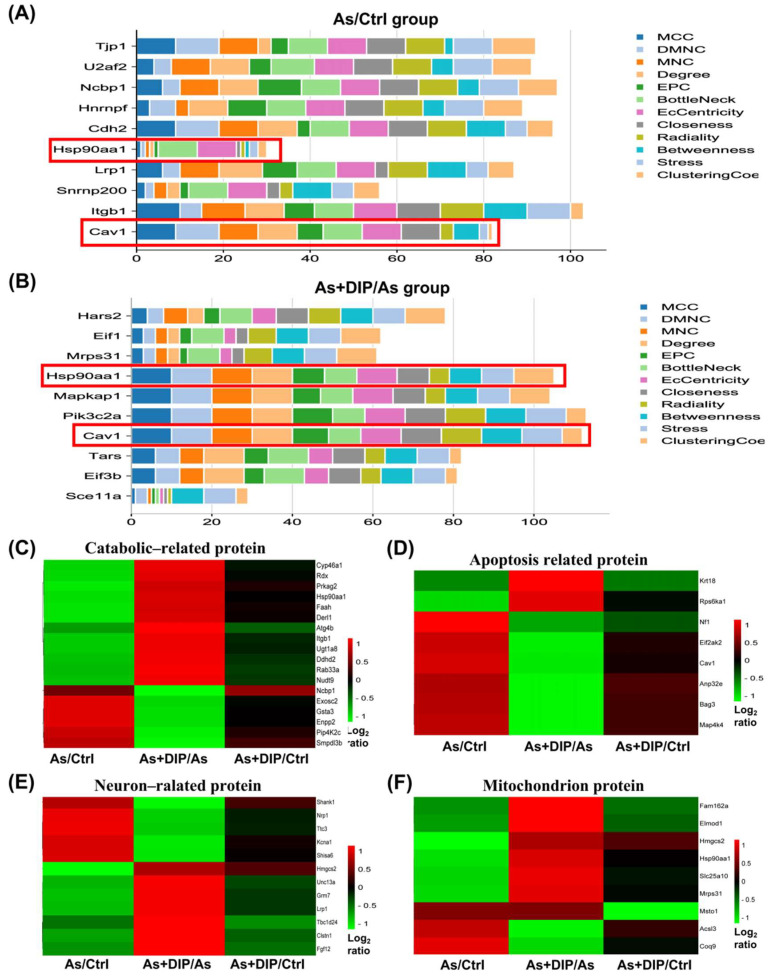
Hub gene and cluster analysis of the differentially expressed proteins in the key pathways in different groups. (**A**) The horizontal stack bar charts the top ten hub genes associated with the As/Ctrl group based on 12 hybrid algorithms. (**B**) The horizontal stack bar charts the top ten hub genes associated with the DIP + As/As group based on 12 hybrid algorithms. (**C**) Cluster analysis of catabolic-related proteins. (**D**) Cluster analysis of apoptosis-related proteins. (**E**) Cluster analysis of neuron-related proteins. (**F**) Cluster analysis of mitochondrial proteins.

Cytohubba software was used to analyze the hub genes in the As/Ctrl and DIP + As/As groups. The top ten hub genes in the As/Ctrl group were identified in Figure 8A. Among them, Cav1 and Hsp90aa1 belonged to reversed proteins. The top ten hub genes in the DIP + As/As group were also identified in Figure 8B. Among these, Cav1, Eif1, Eif3b, Hars2, Hsp90aa1, and Mrps31 belonged to reversed proteins. Evidently, Cav1 and Hsp90aa1 were common hub genes in these two groups. These hub genes were associated with catabolic, apoptosis, mitochondrion, and neuron.

According to UniProt annotation and GO analysis results, we further clustered and analyzed the expressions of DEPs in the key pathways (catabolic, neuron, and apoptosis-related protein, mitochondrial protein) in different groups. The results showed that their expression was reversed or restored after treatment with DIP (Figure 8C–F).

## 3. Discussion

Arsenite exposure impaired the spatial learning and memory ability of rodents have been reported in several previous studies [12,13]. Consistently, here, our results showed that arsenic exposure could damage the spatial learning and memory ability of SD rats. However, DIP treatment could improve the learning and memory ability of arsenic-exposed rats.

Through proteomics analysis, the results showed that the DEPs in the As/Ctrl groups were mainly associated with the synapse, neuron part, apoptosis, mitochondrial and energy metabolism, and catabolic process. Among them, 16 DEPs belonged to synaptic proteins, 19 DEPs belonged to the neuron part, and four DEPs were related to synaptic transmission. These results suggest that arsenic treatment dysregulated the expression of synapses and neuron-related proteins in the hippocampus of SD rats, thus contributing to the impairment of learning and memory in rats exposed to arsenic. This is similar to previous research where arsenic exposure disrupted synapse-related protein expression and signals [14].

Glutamate is the major excitatory neurotransmitter in the mammalian central nervous system and plays an important role in spatial learning and memory function [12]. We found that the expression of several proteins associated with it in the As/Ctrl groups had changed. For example, the expression of Unc13a and GRM7 were downregulated in the hippocampus of arsenic-treated SD rats. Unc13a is essential for synaptic vesicle maturation in most excitatory/glutamatergic that enhances neurotransmitter release [15]. Grm7, as the G-protein coupled receptor activated by glutamate, is related to neurodevelopmental disorders [16]. Therefore, dysregulation of glutamate related proteins might inhibit the growth of glutamatergic and reduce the release of neurotransmitters, which may be responsible for the learning and memory impairment of arsenic-exposed rats. The expression of these synaptic and neuron-related proteins was reversed or restored in the DIP + As/Ctrl group, indicating that DIP improved spatial memory and exploring ability in arsenic-exposed SD rats. Furthermore, in the DIP + As/As group, 13 DEPs were associated with nervous system development, implying that the protective effect of DIP was related to synaptic and neuron-related proteins.

In addition, in the As/Ctrl group, 10 DEPs were found to be associated with regulation of the apoptotic process, suggesting that apoptosis might be one of the important mechanisms of arsenic toxicity in rat hippocampus. Among these DEPs, Krt18 was a regulator of neuronal apoptosis. Eif2ak2 plays a role in apoptosis, cell proliferation, and differentiation. It regulates various signaling pathways (p38 MAP kinase, NF-kappa-B, and insulin signaling pathways). Bag3 is involved in the regulation of the extrinsic apoptotic signaling pathway. Cav1 is a scaffold protein in the cell membrane, which is closely related to cell apoptosis. The expression of these proteins was increased in the As/Ctrl group, which may be related to the apoptosis of hippocampal cells induced by NaAsO_2_. On the other hand, Krt18 plays a role in the negative regulation of apoptotic process. Rps6ka1 functions as a differentiation factor by modulating mTOR signaling and suppressing the pro-apoptotic functions of BAD and DAPK1. Their downregulation in the As/Ctrl group may also contribute to NaAsO_2_-induced apoptosis. Interestingly, Krt18 [17], Cav1 [18], and Bag [19] have been reported to be associated with arsenic-induced apoptosis. In the current study, Cav1 was also identified as a common Hub protein in the As/Ctrl group and the As DIP + As/As group.

Previous studies have shown that arsenic could induce neuronal cell death through oxidative stress [20]. Arsenic toxicity can increase the level of ROS in organs, cause damage to the mitochondrial respiratory chain, disrupt glucose homeostasis in tissues, and induce oxidative stress [21] as well as lead to ROS-induced apoptosis [22]. As expected, in this study, the expression of 29 mitochondrial-associated proteins in the hippocampus of SD rats altered after arsenic treatment. However, their expression was reversed in the DIP + As/As group. The results suggest that they may be related to the neurotoxicity of arsenic and responsible for DIP attenuating arsenic-induced mitochondrial function damage. Indeed, in our previous proteomics study on hepatocytes treated with arsenic, the proteins related to apoptosis and mitochondria were the most dysregulated proteins, while pretreatment with DIP reversed or restored the expression of those proteins [23].

One of the main functions of mitochondria is related to energy metabolism. In this study, catabolic pathways were enriched including the ribonucleotide catabolic process, ascorbate and aldarate metabolism, pyruvate metabolism, and glycosaminoglycan degradation. These pathways have been observed in proteomics studies of arsenic-exposed rats [24] or yeast [25]. We found that most of the proteins in these pathways were downregulated in the As/Ctrl group. This indicated that NaAsO_2_ treatment resulted in abnormal catabolic pathways in the hippocampus of SD rats. However, the expression of these DEPs was reversed in the As + DIP/As group, suggesting that DIP reduced the effect of arsenic. In particular, pyruvate is closely related to adenosine triphosphate (ATP). When pyruvate metabolism is inhibited, it may affect the production of ATP by hindering the aerobic decomposition of pyruvate, increasing the consumption of ATP, and eventually reducing the energy levels [26]. Of note, seven DEPs were identified as ATP binding related proteins including Hsp90aa1, Rdx, Pip4K2c, Rab33a, Nudt9, Derl1, and Prkag2. Among them, the expression levels of six DEPs showed a downward trend, while Hsp90aa1 may play a key role. HSP90AA1 has a variety of functions including involvement in ATPase activation, transcriptional regulation, mitochondrial import, cell growth, development, and apoptosis. In this study, it was found to be a common Hub protein in the As/Ctrl group and the As DIP + As/As group.

**Figure 9:**
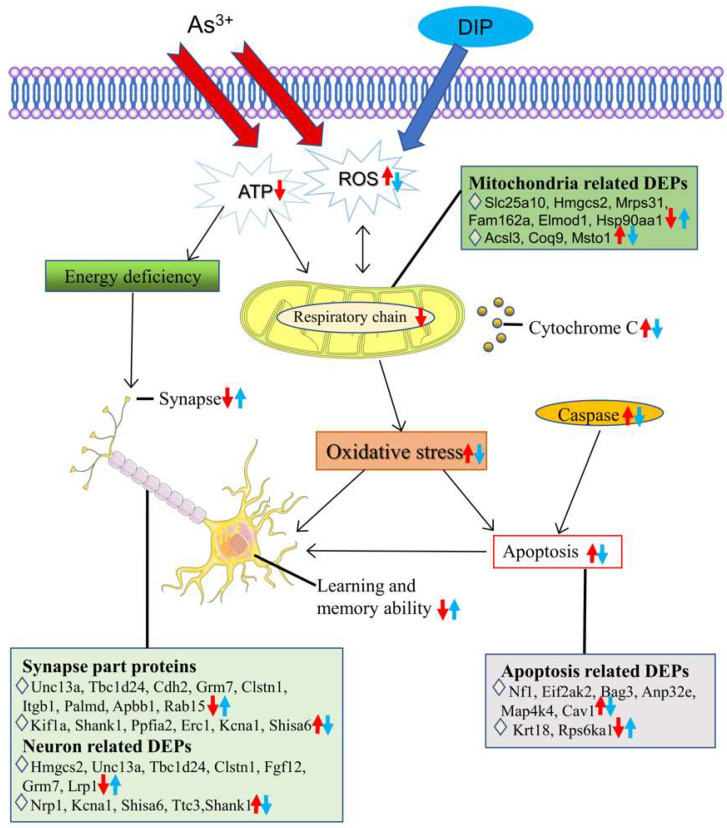
The underlying mechanisms of sodium arsenite exposure impaired learning and memory in SD rats and DIP attenuated this impairment. Arsenic exposure can cause dysregulation of energy metabolism, apoptosis, synapses, neurons, and mitochondria-related proteins in the hippocampus of SD rats. The mechanisms by which these proteins are associated may be interactive. Arsenic induces ROS production, leads to mitochondrial dysfunction, inhibits ATP production, and further causes oxidative stress, ultimately leading to cell apoptosis and synaptic dysfunction. In contrast, DIP reversed or restored the expression of these proteins, helping to improve NaAsO_2_-induced learning and memory impairments in rats. This may be related to the antioxidant effect of DIP. ↑: upregulation. ↓: downregulation. Red is related to As^3+^, blue is related to DIP.

Therefore, our results agree with previous studies that arsenic inhibits ATP production, leading to abnormal energy metabolism. As the main manufactory of cellular ATP, energy-deficiency, mainly resulting from mitochondrial damage, can lead to the dysfunction of synaptic neurotransmission, and further cause nerve injury. DIP has a neuroprotective effect, which can reduce the neurotoxicity of arsenic and improve the nerve injury caused by arsenic. In addition, improved mitochondrial biosynthesis and redox status can alleviate mitochondrial dysfunction. DIP may serve as a potential scavenger for products of oxidative stress products [27], thereby reducing ROS levels to ameliorate mitochondrial dysfunction caused by arsenic. We also found 50 DEPs related to metabolic processes in the DIP + As/Ctrl group, indicating that DIP improved hippocampal metabolism in arsenic-exposed SD rats by being associated with metabolism-related proteins and pathways. Indeed, the relationship between energy-deficiency and nerve injury associated with arsenic has been well reported [28]. However, most of the studies on the effect of arsenic on mitochondrial function have been conducted on cardiac, liver, kidney, and cells. To the best of our knowledge, this is the first study to show that arsenic causes a decrease in the expression of ATP binding proteins and mitochondrial proteins in the hippocampus, resulting in impaired learning and memory function in arsenic-treated SD rats, whereas DIP improved the arsenic-treated SD rats’ learning and memory by reversing or restoring their expression. In addition, we performed subgroup analysis on the sex of rats, and there was no significant difference between the results of each group and the original results. Taken together, the toxic mechanisms of arsenic on energy metabolism, apoptosis, synapse, neuron, and mitochondrion in rat hippocampus and the intervention effect of DIP are summarized in Figure 9.

Moreover, we finally focused on 43 reversed expressions and hub genes, with expression patterns supporting the above discussion. Likewise, we found that some DEPs in the As/Ctrl group were related to the Ras protein signal transduction, small GTPase mediated signal transduction, MAP kinase activity pathway, and Wnt signaling pathway. Most of these DEPs were downregulated in the As/Ctrl group and reversed in the As + DIP/As group. Several studies have shown that arsenic can inhibit the Wnt signaling pathway and affect cell proliferation, differentiation, and apoptosis [29]. Inhibition of the GTPase signaling pathway can induce neuronal apoptosis, which is mediated by a variety of mitogen-activated protein kinase signal cascades [30]. Arsenic has been shown to induce apoptosis in rat neurons by activating p38 MAP kinases [31]. Our results suggest that arsenic-induced neurotoxicity and the improvement in DIP on arsenic induced learning and memory impairment in rats may be related to these signal pathways.

## 4. Materials and Methods

### 4.1. Chemicals and Dictyophora Polysaccharide Preparation

Food-grade Dictyophora was obtained from Zhijin Sifang Hongye Co., Ltd., (Zhijin City, China). Sodium arsenite (NaAsO_2_) was purchased from Sigma Chemical Corp (St. Louis, MO, USA). DIP was prepared in accordance with our previous study [11,23,32]. Briefly, the Dictyophora fruiting body was ground into a powder, extracted, filtered, and concentrated. The crude polysaccharides of Dictyophora were obtained by freeze-drying after overnight precipitation with four volumes of 100% ethanol.

### 4.2. Animals and Experimental Design

The whole work scheme is shown in Figure 1A. Sixty 6-week-old SD rats (30 male and 30 female) were obtained from Huafukang Biotechnology Co., Ltd., (Beijing, China). Rats were fed a standard diet and allowed to drink freely for one week while being kept at a temperature of 20 ± 2 °C and a relative humidity of 55 ± 5%.

After one week of adaptive feeding, they were randomly divided into three groups with 20 rats in each group (half male and half female). The As and DIP concentrations used in the groups were determined according to the previous studies [10,32,33]. The details were as follows: for the control group, common feedstuff and distilled water were given for four months, with physiological saline (0.9 percent NaCl) gavage once a day in the fourth month. For the As group, processed feedstuff with 50 mg/kg NaAsSO_2_ and distilled water were given for four months, with physiological saline (0.9 percent NaCl) gavage once a day in the fourth month. For the As + DIP group, processed feedstuff with 50 mg/kg NaAsSO_2_ and distilled water were given for four months. Gavage bamboo fungus polysaccharide beverage was then administered once a day in the fourth month.

### 4.3. Morris Water Maze (MWM) Test

The MWM test was used to check the spatial learning and memory capacities of rats [34]. Briefly, each rat was trained four times over the course of four days. On the fifth day of the space exploration experiment (SPaceex Plorationex Periment), the platform was removed and the 120 s exploration training began. The following information was recorded as the rats entered: the number of times the rats passed through the target (the original platform’s position) and the swimming time they took to cross the target quadrant (the quadrant where the platform was originally placed).

### 4.4. Sample Preparation for Proteomic Analysis

After four months of treatment, rats were euthanized by cervical dislocation with their brains rapidly excised on an ice-cold plate for hippocampal dissection. Ten rats (five male and five female) were chosen randomly for proteomic analysis in each group. Hippocampal tissue was homogenized by using the Freezing grinder (Jingxin, Shanghai, China) and lysed in RIPA lysis buffer (KeyGEN BioTECH, Nanjing, Jiangsu, China), and centrifuged at 12,000 rpm, 4 °C for 30 min. The supernatants were collected with protein concentrations quantified using the BCA assay (KeyGEN BioTECH) and stored at −80 °C until use. For trypsin digestion, 200 μg protein of each sample was reduced with 10 mM DTT (dithiothreitol) at 37 °C for 2 h before being alkylated with 55 mM iodoacetamide (IAM) at room temperature for 30 min in darkness. After this, it was digested with trypsin at 37 °C overnight. The peptides were dried up in a vacuum centrifuge and stored at 4 °C for LC-MS/MS analysis.

### 4.5. Generation of the Reference Spectral Library by Independent Data Analysis (IDA) Acquisition

In order to perform SWATH-MS analysis, a reference spectral library needs to be established. A mixed representative sample of all samples was obtained by mixing 15 μg of protein from each sample and used for quality control (QC). The liquid chromatography (LC) was carried out by Agilent 1100 Serise liquid phase system (Agilent Technologies, Santa Clara, CA, USA). The conditions for chromatographic separation of protein samples were as follows: flow rate—0.30 mL/min; injection volume—100 μL; mobile phase: phase A, water (containing 0.1% formic acid); phase B, acetonitrile:water (95:5, *v*/*v*); the gradient of liquid phase elution was 70 min, and phase B increased from 5% to 80%: 5% phase B was the initial concentration (0−3 min), 5−38% phase B (3−60 min), 38−80% phase B (60−65 min), and 5% phase B equilibrium to 70 min. The mass spectrometer was a Triple TOF 6600 (SCIEX, Framingham, MA, USA). In the IDA mode, a time-of-flight mass spectrometry (TOF-MS) survey scan was acquired (350–1500 *m*/*z*, 0.25 s) with the 40 most intense multiply charged ions (2+ to 5+; exceeding 100 counts per s). MS/MS spectra were accumulated for 50 ms in the mass range of 100–1500 *m*/*z* with rolling collision energy. To create the library, the MS raw files and databases searches were combined and performed using ProteinPilot software v.5.0.1 (SCIEX).

### 4.6. Quantification of Proteins by SWATH Acquisition

The SWATH-MS acquisition was performed in DIA mode, with the peptides from each sample collected separately after enzymatic hydrolysis. The QC sample was collected first, and subsequent quality control analyses were performed by collecting a QC sample every five samples. Data were acquired with a 2.4 kV ion spray voltage, 35 psi curtain gas, 12 psi nebulizer gas, and an interface heater temperature of 150 °C. The scanning resolution of the primary mass spectrometry was 70,000, the scanning range was 100–1500 *m*/*z*, and the maximum injection time was 40 ms. Each cycle contained 34 distinct windows. The variable width between windows was 80 w, the window mass range was 12.5–158 Da, and the maximum ion implantation time of secondary mass spectrometry was 50 ms. The collision chamber energy (high energy collision induced dissociation, HCD) was set to 28 ev. Accurate quantitation was conducted using MultiQuant™ software. The cutoff values were 1.5-fold for upregulated and 0.67-fold for downregulated proteins.

### 4.7. Statistical Analysis

The MWM test data from the three groups were subjected to one-way ANOVA analysis. Previously, normal distribution and variance homogeneity were investigated (Shapiro–Wilk). Statistical analysis was carried out using the SPSS package (SPSS 23.0, Chicago, IL, USA).

### 4.8. Bioinformatics Analysis

The data were corrected using the R language software statTarget tool based on QC samples. Heatmap was plotted on an online platform for data analysis and visualization (http://www.bioinformatics.com.cn, accessed on 7 October 2021). Bioinformatics analysis was performed using OMICSBEAN online tools (http://www.omicsbean.cn, accessed on 1 October 2021) and the String database (http://string-db.org, version 9.1, accessed on 15 September 2021). DEPs were analyzed by GO (gene ontology) annotation, KEGG (Kyoto Encyclopedia of Genes and Genomes) pathways, and protein−protein interaction (PPI) networks. GO annotations included the biological process (BP) and cellular component (CC) [35]. Hub genes were screened by cytohubba software.

## 5. Conclusions

In this study, we showed that NaAsO_2_ induced learning and memory impairments in SD rats. Nevertheless, DIP treatment restored their learning and memory. Changes in energy metabolism, apoptosis, synapse, neuron, and mitochondrial related proteins were observed in the hippocampus of arsenic induced rats. Their changes led to mitochondrial dysfunction, abnormal energy metabolism, apoptosis, and abnormal synaptic structure and neurotransmitter, resulting in learning and memory impairment of As induced rats. Several pathways including the Ras protein signal transduction, small GTPase mediated signal transduction, MAP kinase activity pathway, and Wnt signaling pathway may also play an important role. Importantly, this is the first study to observe that arsenic induces downregulation of ATP binding and mitochondrial-related protein expression in the hippocampus of rats. The expression of these proteins was reversed by DIP treatment. This is also the first study to link DIP to arsenic-induced apoptosis and neurotoxicity. These findings may provide a direction for arsenic neurotoxicity research using DIP treatment. Future studies are needed to validate the molecular mechanisms of arsenic-induced hippocampal toxicity and the implications presented by the findings in the current study.

## Figures and Tables

**Table 1:** Reversed proteins Identified in the present study.

No.	Protein Name	Gene Name	Uniprot Assention	As/Ctrl	As + DIP/As	As + DIP/Ctrl
1	Prefoldin 5 (Predicted), isoform CRA_a	Pfdn5	B5DFN4	**0.47**a	**0.57**a	**0.27**a
2	Protein PRRC1	Prrc1	G3V834	**0.21**a	**2.87**a	0.61
3	Histidine--tRNA ligase	Hars2	F1M9C9	**0.26**a	**3.36**a	0.89
4	Family with sequence similarity 219, member A	Fam219a	D4AAI7	**0.33**a	**1.82**a	0.60
5	Ribonuclease L	Rnasel	G3V915	**0.43**a	**2.16**a	0.93
6	Ribosomal protein S6 kinase	Rps6ka1	F1LXV0	**0.45**a	**1.97**a	0.88
7	Nicotinamide phosphoribosyltransferase	Nampt	A0A0G2K0I3	**0.45**a	**1.71**a	0.77
8	Derlin	Derl1	F7FNS3	**0.46**a	**2.55**a	1.17
9	Protein misato homolog 1	Msto1	D3ZMW3	**0.46**a	**1.64**a	0.76
10	28S ribosomal protein S31, mitochondrial	Mrps31	B0BN56	**0.47**a	**1.92**a	0.91
11	Cyp46a1 protein	Cyp46a1	F7EN52	**0.50**a	**1.71**a	0.85
12	Radixin	Rdx	E9PT65	**0.52**a	**2.69**a	1.39
13	Mitochondrial dicarboxylate carrier	Slc25a10	O89035	**0.53**a	**1.62**a	0.86
14	PiggyBac transposable element derived 5 (Predicted)	Pgbd5	D3ZSZ4	**0.53**a	**1.67**a	0.89
15	Sorbin and SH3 domain-containing protein 2	Sorbs2	F1LPM3	**0.55**a	**1.80**a	1.00
16	BolA family member 2	Bola2	D4A9P7	**0.60**a	**1.56**a	0.93
17	Protein Dr1	Dr1	Q5XI68	**0.61**a	**1.57**a	0.95
18	Heat shock protein HSP 90-alpha	Hsp90aa1	P82995	**0.62**a	**1.68**a	1.03
19	Eukaryotic translation initiation factor 3 subunit B	Eif3b	Q4G061	**0.62**a	**1.54**a	0.96
20	Eukaryotic translation initiation factor 1	Eif1	B0K008	**0.64**a	**1.59**a	1.02
21	Fatty-acid amide hydrolase 1	Faah	P97612	**0.65**a	**1.72**a	1.11
22	Regulator of microtubule dynamics protein 3	Rmdn3	Q66H15	**0.65**a	**1.60**a	1.04
23	Signal peptidase complex catalytic subunit SEC11	Sec11a	Q6P9X2	**0.66**a	**1.61**a	1.07
24	E3 ubiquitin-protein ligase TTC3	Ttc3	D3ZSP7	**1.57**a	**0.50**a	1.16
25	Shisa family member 6	Shisa6	D4A4M0	**1.60**a	**0.66**a	1.06
26	BCS1-like protein	Bcs1l	Q5XIM0	**1.65**a	**0.65**a	1.07
27	LIM and calponin homology domains 1	Limch1	F1M392	**1.69**a	**0.52**a	0.88
28	4-trimethylaminobutyraldehyde dehydrogenase	Aldh9a1	A0A0G2JSI1	**1.71**a	**0.62**a	1.07
29	Ubiquinone biosynthesis protein COQ9, mitochondrial	Coq9	Q68FT1	**1.72**a	**0.53**a	0.91
30	MAP7 domain-containing 2	Map7d2	D4A4L4	**1.72**a	**0.54**a	0.92
31	MTSS I-BAR domain-containing 2	Mtss1l	D4A3S6	**1.80**a	**0.54**a	0.97
32	Proline-rich transmembrane protein 3	Prrt3	D3ZWQ0	**1.82**a	**0.61**a	1.10
33	PTPRF-interacting protein alpha 2	Ppfia2	F1M8A4	**1.82**a	**0.46**a	0.84
34	Pleckstrin and Sec7 domain-containing 3	Psd3	D4A2Q3	**1.83**a	**0.66**a	1.21
35	Caveolin	Cav1	Q2IBC6	**1.88**a	**0.63**a	1.18
36	ER membrane protein complex subunit 10	Emc10	Q6AYH6	**1.89**a	**0.66**a	1.24
37	Ectonucleotide pyrophosphatase/phosphodiesterase family member 2	Enpp2	Q64610	**1.97**a	**0.51**a	1.00
38	Glutathione S-transferase alpha-3	Gsta3	P04904	**2.07**a	**0.49**a	1.02
39	Ribosomal RNA-processing protein 4	Exosc2	D3ZBP3	**2.11**a	**0.44**a	0.94
40	Potassium voltage-gated channel subfamily A member 1	Kcna1	P10499	**2.30**a	**0.53**a	1.21
41	Hydroxymethylbilane hydrolyase [cyclizing]	Uros	Q5XIF2	**2.41**a	**0.62**a	1.51
42	Long-chain-fatty-acid--CoA ligase 3	Acsl3	Q63151	**2.50**a	**0.63**a	1.57
43	Uridine-cytidine kinase	Uckl1	D3ZYQ8	**2.95**a	**0.33**a	0.96
44	Protein arginine methyltransferase NDUFAF7, mitochondrial	Ndufaf7	Q5XI79	**3.71**a	**0.37**a	1.38
45	Uncharacterized protein	Dock2	F7F7H4	**0.15**a	2.56	**0.38**a
46	RAB33A, member RAS oncogene family	Rab33a	D3ZCU8	**0.26**a	1.72	**0.45**a
47	Ubiquitin carboxyl-terminal hydrolase	Fam63b	D3ZWA1	**0.27**a	1.15	**0.31**a
48	Mitogen-activated protein kinase kinase kinase 2	Map4k2	D3ZXB1	**0.32**a	0.92	**0.30**a
49	Metallo-beta-lactamase domain-containing 2	Mblac2	D4A249	**0.33**a	1.33	**0.44**a
50	Hypothetical protein MGC:15854	RGD1302996	G3V628	**0.34**a	1.36	**0.46**a
51	Alpha-MPP	Pmpca	Q68FX8	**0.37**a	1.34	**0.50**a
52	Microtubule associated protein 11	RGD1305455	A0A0G2KAX2	**0.40**a	0.85	**0.34**a
53	Keratin, type I cytoskeletal 18	Krt18	Q5BJY9	**0.43**a	1.05	**0.45**a
54	Calmin	Clmn	D4A626	**0.44**a	1.02	**0.45**a
55	Tight junction protein ZO-1	Tjp1	A0A0G2K2P5	**0.45**a	1.10	**0.49**a
58	Solute carrier family 7, member 14	Slc7a14	A0A0G2K1G8	**0.47**a	1.28	**0.61**a
59	CD151 antigen	Cd151	Q9QZA6	**0.47**a	0.87	**0.41**a
60	Cysteine protease	Atg4b	A0A0G2QC33	**0.48**a	1.14	**0.54**a
61	HECT-type E3 ubiquitin transferase	Ube3c	D3ZHB7	**0.48**a	0.87	**0.42**a
62	Proline-rich protein 7	Prr7	P0C6T3	**0.48**a	0.70	**0.34**a
63	Dystrophin	Dmd	Q7TPH4	**0.50**a	1.04	**0.52**a
64	Inosine-5’-monophosphate dehydrogenase 2	Impdh2	E9PU28	**0.52**a	1.20	**0.62**a
65	Amyloid-beta A4 precursor protein-binding family B member 1	Apbb1	P46933	**0.56**a	1.09	**0.61**a
66	TBC1 domain family member 24	Tbc1d24	D4A3Z3	**0.56**a	0.97	**0.54**a
67	Transmembrane protein 151A	Tmem151a	M0RAG0	**0.56**a	1.08	**0.61**a
68	Kelch-like protein 22	Klhl22	A0A0G2KA06	**0.57**a	0.75	**0.43**a
69	Protein FAM162A	Fam162a	Q4QQV3	**0.57**a	1.06	**0.61**a
70	SCY1-like pseudokinase 2	Scyl2	D4A1Y0	**0.58**a	1.05	**0.61**a
71	Ras GTPase-activating protein 2	Rasa2	Q63713	**0.61**a	0.69	**0.42**a
72	Chromatin-modifying protein 4B-like 1	Chmp4bl1	D4A9Z8	**0.62**a	0.81	**0.50**a
73	Prostamide/prostaglandin F synthase	Fam213b	D3ZVR7	**0.64**a	0.94	**0.61**a
74	Isochorismatase domain-containing protein 1	LOC103694869	F2Z3T7	**0.65**a	1.02	**0.66**a
75	RCG43947	Txndc5	D3ZZC1	**0.66**a	0.79	**0.52**a
76	Calpain-5	Capn5	G3V7U6	**0.66**a	0.99	**0.65**a
77	Phosphatidic acid phosphatase type 2B	Plpp3	Q6IMX4	**0.66**a	0.90	**0.60**a
78	Similar to RIKEN cDNA 1110063G11 (Predicted)	Tmcc2	D3ZE26	**0.66**a	0.87	**0.57**a
79	Capping protein regulator and myosin 1 linker 2	Carmil2	D3ZC15	**1.68**a	0.93	**1.57**a
80	Proton myo-inositol cotransporter	Slc2a13	Q921A2	**1.69**a	1.24	**2.10**a
81	Retinoid-inducible serine carboxypeptidase	Scpep1	Q920A6	**1.72**a	0.90	**1.56**a
82	ELKS/Rab6-interacting/CAST family member 1	Erc1	A0A0G2JYT1	**1.74**a	0.96	**1.68**a
83	ASPSCR1 tether for SLC2A4, UBX domain-containing	Aspscr1	F1LR71	**1.78**a	1.13	**2.00**a
84	Hemoglobin subunit beta-1	Hbb	P02091	**1.89**a	0.81	**1.53**a
85	Hemoglobin subunit beta-2	ENSRNOG00000031230	P11517	**1.92**a	0.84	**1.61**a
86	Nuclear cap-binding protein subunit 1	Ncbp1	Q56A27	**2.06**a	1.06	**2.17**a
87	Acid sphingomyelinase-like phosphodiesterase	Smpdl3b	Q4V7D9	**2.23**a	0.73	**1.62**a
88	SH3 and multiple ankyrin repeat domains protein 1	Shank1	Q9WV48	**2.33**a	0.74	**1.73**a
89	Proteasome inhibitor PI31 subunit	Psmf1	F1M7S2	**2.45**a	0.89	**2.17**a
90	YTH N(6)-methyladenosine RNA-binding protein 1	Ythdf1	Q4V8J6	**2.50**a	1.14	**2.87**a
91	Spermine synthase	Sms	Q3MIE9	**3.36**a	0.92	**3.07**a
92	Serine/threonine-protein kinase PRP4 homolog	Prpf4b	Q5RKH1	**4.79**a	0.64	**3.07**a
93	Fam81a	Fam81a	D4A7T8	0.83	**0.60**a	**0.50**a
94	40S ribosomal protein S15	Rps15	P62845	1.03	**0.60**a	**0.58**a
95	Ras suppressor protein 1	Rsu1	D4A8F2	0.84	**1.82**a	**1.52**a
96	Target of rapamycin complex 2 subunit MAPKAP1	Mapkap1	Q6AYF1	1.08	**1.56**a	**1.68**a
97	CaM kinase-like vesicle-associated protein	Camkv	A0A0G2K1R5	0.93	**2.81**a	**2.66**a

a Log2 Fold change, in bolded text, *p* < 0.05.

## Data Availability

The data presented in this study are available on request.

## Supplementary Materials

The following supporting information can be downloaded at, Table S1: Fifteen important biological process in GO analysis in the As/Ctrl group, Table S2: Fifteen important cellular component in GO analysis in the As/Ctrl group, Table S3: KEGG pathways associated with the differentially expressed proteins in the As/Ctrl group, Table S4: Fifteen important biological process in GO analysis in the As + DIP/As group, Table S5: Fifteen important cellular component in GO analysis in the As + DIP/As group, Table S6: KEGG pathways associated with the differentially expressed proteins in the As + DIP/As group, Table S7: Ten important biological process associated with the reversed proteins in GO analysis, Table S8: The pathways associated with the reversed proteins in KEGG analysis.
