# Supplementary material for: Investigating the Neurotoxic Impacts of Arsenic and the Neuroprotective Effects of Dictyophora Polysaccharide Using SWATH-MS-Based Proteomics
Source: Molecules. 2022 Feb 23;27(5):1495. doi: 10.3390/molecules27051495 (PMC8911851; doi:10.3390/molecules27051495)
Supplement: Supplementary file 1 [file molecules-27-01495-s001.zip › molecules-1607068-supplementary.pdf]

Table S1 15 important biological process (BP) in GO analysis in the As/Ctrl group

GO Name	GO ID	Genes	Count
mitochondrion organization	GO:0007005	Ddhd2,Pmpca,Bcs1l,Ndufaf7,Slc25a46,Hars2,Elmod1,Tfam,U2af2,Bag3,Fam162a,Msto1	12
neuromuscular process	GO:0050905	Shank1,Kcna1,Fgf12,Rbfox1	4
cellular catabolic process	GO:0044248	Lrp1,Hsp90aa1,Ube3c,Ddhd2,Faah,Pde10a,Gsta3,Rdx,Ncbp1,Pip4k2c,Atg4b,Rab33a,U2af2,Nudt9,Smpdl3b,Psmf1,Exosc2,Scpep1,Ttc3,Derl1,Ugt1a8,Enpp2	22
negative regulation of Wnt signaling pathway	GO:0030178	Cdh2,Shisa6,Pfdn5,Cav1	4
catabolic process	GO:0009056	Lrp1,Hsp90aa1,Cyp46a1,Ube3c,Ddhd2,Faah,Pde10a,Itgb1,Gsta3,Rdx,Ncbp1,Pip4k2c,Atg4b,Rab33a,U2af2,Nudt9,Smpdl3b,Psmf1,Exosc2,Scpep1,Ttc3,Derl1,Ugt1a8,Enpp2	24
organophosphate catabolic process	GO:0046434	Pde10a,Nudt9,Smpdl3b,Enpp2	4
regulation of small GTPase mediated signal transduction	GO:0051056	Arfgef3,Psd3,Cdh2,Itgb1,Arhgef17,Rasa2	6
single-organism catabolic process	GO:0044712	Cyp46a1,Ddhd2,Faah,Pde10a,Itgb1,Atg4b,Rab33a,U2af2,Nudt9,Smpdl3b,Derl1,Ugt1a8,Enpp2	13
regulation of mitochondrion organization	GO:0010821	Ddhd2,Elmod1,U2af2,Bag3,Fam162a	5
positive regulation of synaptic transmission	GO:0050806	Unc13a,Shank1,Tbc1d24,Clstn1	4
obsolete cell-type specific apoptotic process	GO:0097285	Lrp1,Hsp90aa1,Krt18,Bag3,Fam162a,Cav1	6
negative regulation of Ras protein signal transduction	GO:0046580	Itgb1,Rasa2	2
regulation of MAP kinase activity	GO:0043405	Eif2ak2,Map4k4,Lamtor3,Map4k2,Cav1	5
negative regulation of apoptotic process	GO:0043066	Lrp1,Krt18,Grm7,Itgb1,Eif2ak2,Bag3,Rps6ka1,Map4k4,Anp32b,Cav1	10
regulation of translational initiation	GO:0006446	Eif3b,Fech,Ncbp1,Habp4,Eif2ak2,Ythdf1	6

Table S2 15 important cellular component (CC) in GO analysis in the As/Ctrl group

GO_Name	GO_ID	Genes	Count
cytoplasmic part	GO:0044444	Lrp1,Sptb,Hsp90aa1,Kif1a,Copa,Unc13a,Shank1,Tjp1,Arfgef3,Tomm70,Exoc2,Eif3b,Erc1,Acsl3,Acss1,Cdh2,Hbb,Gna13,Krt18,Grm7,Ddhd2,Carmil2,Pmpca,Impdh2,Grhpr,Aimp1,Bcs1l,Faah,Clstn1,Itgb1,Ndufaf7,Rmdn3,Kpna4,Kcna1,Slc25a46,Rbsn,Bcl2l13,Tmcc2,Gsta3,Aspscr1,Clvs1,Hars2,Fech,Pmm1,Rdx,Ncbp1,Slc25a10,Pip4k2c,Mtss1l,Atg4b,Apbb1,Hnrnpf,Txndc5,Rexo2,Rab33a,Plpp3,Elmod1,Habp4,Fam213b,Rab27b,Rab15,Tfam,Eif2ak2,Aimp2,Nudt9,Mrps31,Coq9,Psmf1,Bag3,Rps6ka1,Anp32e,Uckl1,Fam162a,Tmem33,Dhrs4,Msto1,Exosc2,Slc16a1,Rbfox1,Scpep1,Ttc3,Mrpl40,Pfdn2,Sec11a,Chmp3,RGD1302996,Uros,Lamtor3,Emc10,ENSRNOG00000031230,Cav1,Derl1,Mrps33,Ugt1a8,Jagn1,Prr7,Enpp2	97
intracellular part	GO:0044424	Lrp1,Sptb,Hsp90aa1,Kif1a,Snrnp200,Copa,Unc13a,Shank1,Tjp1,Arfgef3,Tomm70,Exoc2,Ipo4,Eif3b,Erc1,Tbc1d24,Acsl3,Man2c1,Clmn,Ube3c,Capn5,Acss1,Sorbs2,Cdh2,Hbb,Gna13,Krt18,Klhl22,Grm7,Ddhd2,Carmil2,Pmpca,Impdh2,Grhpr,Aimp1,Bcs1l,Faah,Clstn1,Pde10a,Itgb1,Ndufaf7,Rmdn3,Kpna4,Kcna1,Slc25a46,Map7d2,Rbsn,Bcl2l13,Tmcc2,Shisa6,Gsta3,Aspscr1,Clvs1,Hars2,Cpne1,Palmd,Fech,Pmm1,Rdx,Ncbp1,Slc25a10,Pip4k2c,Mtss1l,Atg4b,Apbb1,RGD1305455,Hnrnpf,Txndc5,Pfdn5,Rexo2,Rab33a,Plpp3,Elmod1,Habp4,Fam213b,Rab27b,Rab15,Tfam,U2af2,Eif2ak2,Aimp2,Nudt9,Mrps31,Coq9,Nt5c3b,Psmf1,Bag3,Fam63b,Rps6ka1,Anp32e,Uckl1,Fam162a,Tmem33,Dhrs4,Msto1,Exosc2,Fgf12,Slc16a1,Rbfox1,Map4k4,Scpep1,Ttc3,Mrpl40,Phactr3,Rasa2,Prpf4b,Pfdn2,Anp32b,Sec11a,Chmp3,Gng7,Srsf4,Msi2,Rbm22,RGD1302996,Uros,Lamtor3,Map4k2,Rpap1,Emc10,ENSRNOG00000031230,Rnasel,Cav1,Derl1,Mrps33,Ugt1a8,Dr1,B9d2,Jagn1,Prr7,Enpp2	131

organelle	GO:0043226	Lrp1,Sptb,Hsp90aa1,Kif1a,Snrnp200,Copa,Shank1,Tjp1,Arfgef3,Tomm70,Ppfia2,Ipo4,Eif3b,Acsl3,Man2c1,Capn5,Acss1,Sorbs2,Aldh9a1,Cdh2,Gna13,Krt18,Klhl22,Grm7,Ddhd2,Carmil2,Pmpca,Impdh2,Grhpr,Sms,Bcs1l,Faah,Clstn1,Itgb1,Ndufaf7,Rmdn3,Kpna4,Kcna1,Slc25a46,Map7d2,Rbsn,Bcl2l13,Tmcc2,Shisa6,Atp6ap1,Gsta3,Aspscr1,Clvs1,Hars2,Cpne1,Mblac2,Fech,Rdx,Ncbp1,Slc25a10,Pip4k2c,Mtss1l,Apbb1,RGD1305455,Hnrnpf,Txndc5,Rexo2,Rab33a,Plpp3,Elmod1,Habp4,Fam213b,Rab27b,Rab15,Tfam,U2af2,Aimp2,Nudt9,Smpdl3b,Mrps31,Coq9,Psmf1,Bag3,Fam63b,Rps6ka1,Anp32e,Fam162a,Tmem33,Dhrs4,Msto1,Exosc2,Fgf12,Slc16a1,Rbfox1,Scpep1,Ttc3,Mrpl40,Phactr3,Prpf4b,Pfdn2,Nudt14,Anp32b,Sec11a,Chmp3,Srsf4,Rbm22,RGD1302996,Uros,Lamtor3,Rpap1,Emc10,Rnasel,Cav1,Derl1,Mrps33,Dock2,Ugt1a8,Dr1,B9d2,Jagn1,Prr7,Enpp2	117
mitochondrion	GO:0005739	Tomm70,Acsl3,Acss1,Pmpca,Bcs1l,Ndufaf7,Rmdn3,Slc25a46,Bcl2l13,Hars2,Fech,Ncbp1,Slc25a10,Rexo2,Tfam,Nudt9,Mrps31,Coq9,Fam162a,Dhrs4,Msto1,Slc16a1,Ttc3,Mrpl40,Pfdn2,RGD1302996,Uros,Cav1,Mrps33	29
synapse part	GO:0044456	Kif1a,Unc13a,Shank1,Erc1,Tbc1d24,Cdh2,Grm7,Clstn1,Itgb1,Kcna1,Shisa6,Palmd,Apbb1,Rab15,Prr7	15
adherens junction	GO:0005912	Lrp1,Tjp1,Capn5,Cdh2,Gna13,Krt18,Itgb1,Rdx,Lamtor3,Cav1	10
synapse	GO:0045202	Kif1a,Unc13a,Shank1,Ppfia2,Erc1,Tbc1d24,Cdh2,Grm7,Clstn1,Itgb1,Kcna1,Shisa6,Palmd,Apbb1,Rab15,Prr7	16
excitatory synapse	GO:0060076	Unc13a,Shank1,Shisa6	3
peroxisomal membrane	GO:0005778	Acsl3,Impdh2,Cav1	3
mitochondrial matrix	GO:0005759	Acss1,Pmpca,Rexo2,Tfam,Mrps31,Mrpl40	6
mitochondrial part	GO:0044429	Tomm70,Acsl3,Acss1,Pmpca,Rmdn3,Slc25a46,Slc25a10,Rexo2,Tfam,Mrps31,Coq9,Msto1,Mrpl40	13
neuron part	GO:0097458	Lrp1,Hsp90aa1,Kif1a,Unc13a,Shank1,Erc1,Tbc1d24,Grm7,Clstn1,Pde10a,Itgb1,Kcna1,Palmd,Pmm1,Rdx,Apbb1,Rab15,Bag3,Prr7	19
neuron to neuron synapse	GO:0098984	Shank1,Cdh2,Grm7,Clstn1,Shisa6	5
ribonucleoprotein complex	GO:1990904	Snrnp200,Ncbp1,Hnrnpf,Habp4,U2af2,Mrps31,Rps6ka1,Mrpl40,Prpf4b,Msi2,Rbm22,Derl1,Mrps33	13

neuron spine	GO:0044309	Shank1,Itgb1,Palmd,Apbb1	4

Table S3 KEGG pathways associated with the DEPs in the As/Ctrl group

Pathway Name	Pathway ID	Genes	Count
Porphyrin and chlorophyll metabolism	rno00860	Fech,Uros,Ugt1a8	3
Spliceosome	rno03040	Snrnp200,Ncbp1,U2af2,Srsf4,Rbm22	5
Drug metabolism - other enzymes	rno00983	Impdh2,Uckl1,Ugt1a8	3
Pyruvate metabolism	rno00620	Acss1,Grhpr	2
Ascorbate and aldarate metabolism	rno00053	Aldh9a1,Ugt1a8	2
Ether lipid metabolism	rno00565	Plpp3,Enpp2	2
Glutamatergic synapse	rno04724	Shank1,Grm7,Gng7	3
Propanoate metabolism	rno00640	Acss1,Aldh9a1	2

Table S4 15 important biological process (BP) in GO analysis in the As+DIP/As group

GO_Name	**GO_ID**	**Genes**	**Count**
negative regulation of canonical Wnt signaling pathway	GO:0090090	Shisa6,Pfdn5,Cav1	3
catabolic process	GO:0009056	Hsp90aa1,Ptpn23,Cyp46a1,Prkag2,Faah,Gsta3,Rdx,Stx12,Ctsa,Exosc2,Ttc3,Trim21,Derl1,Enpp2	14
metabolic process	GO:0008152	Nf1,Hsp90aa1,Atp2b2,Tars,Ptpn23,Eif3b,Nampt,Acsl3,Cyp46a1,Psd3,Aldh9a1,Prkag2,Kif21b,Purb,Tbce,Faah,Nrp1,Ndufaf7,Fkbp5,Gsta3,Hars2,Rdx,Crkl,Stx12,Hmgcs2,Pfdn5,Ctsa,Msra,Coq9,Eif1,Rps6ka1,Rsu1,Uckl1,Msto1,Exosc2,Ttc3,Rabif,Sec11a,Pik3c2a,Uros,Rps15,Galns,Trim21,Rnasel,Cav1,Derl1,Mrps21l,Dr1,Camkv,Enpp2	50
cellular catabolic process	GO:0044248	Hsp90aa1,Ptpn23,Faah,Gsta3,Rdx,Stx12,Ctsa,Exosc2,Ttc3,Trim21,Derl1,Enpp2	12
central nervous system development	GO:0007417	Nf1,Atp2b2,Acsl3,Nrp1,Kcna1,Hmgcs2,Mapkap1,Enpp2	8
negative regulation of neurogenesis	GO:0050768	Nf1,Nrp1,Ttc3,Cav1	4
negative regulation of nervous system development	GO:0051961	Nf1,Nrp1,Ttc3,Cav1	4
extrinsic apoptotic signaling pathway	GO:0097191	Nf1,Nrp1,Cav1	3
phosphorus metabolic process	GO:0006793	Nf1,Atp2b2,Ptpn23,Nampt,Acsl3,Prkag2,Nrp1,Ndufaf7,Crkl,Coq9,Rps6ka1,Uckl1,Pik3c2a,Rnasel,Cav1,Camkv,Enpp2	17
nucleoside phosphate metabolic process	GO:0006753	Nf1,Atp2b2,Nampt,Prkag2,Ndufaf7,Coq9,Uckl1	7
nervous system development	GO:0007399	Nf1,Hsp90aa1,Atp2b2,Acsl3,Nrp1,Kcna1,Shisa6,Crkl,Hmgcs2,Ttc3,Cav1,Mapkap1,Enpp2	13
muscle cell apoptotic process	GO:0010657	Hsp90aa1,Cav1	2
negative regulation of neuron differentiation	GO:0045665	Nrp1,Ttc3,Cav1	3
regulation of hydrolase activity	GO:0051336	Nf1,Psd3,Rdx,Mtss1l,Rps6ka1,Rsu1,Rabif,Cav1	8
translational initiation	GO:0006413	Eif3b,Eif1	2

Tabel S5 15 important cellular component (CC) in GO analysis in the As+DIP/As group

GO Name	GO ID	Genes	Count
cytoplasmic part	GO:0044444	Hsp90aa1,Atp2b2,Tars,Ptpn23,Eif3b,Acsl3,Kif21b,Bcs1l,Faah,Nrp1,Prrc2a,Ndufaf7,Rmdn3,Fkbp5,Kcna1,Gsta3,Hars2,Rdx,Crkl,Stx12,Slc25a10,Mtss1l,Hmgcs2,Ctsa,Dnajc16,Lman1,Msra,Mrps31,Tmem43,Coq9,Rps6ka1,Uckl1,Msto1,Exosc2,Emc7,Ttc3,Rabif,Sec11a,Uros,Rps15,Galns,Emc10,Tmed5,Cav1,Derl1,Mapkap1,Mrps21l,Enpp2	48
cytoplasm	GO:0005737	Nf1,Hsp90aa1,Atp2b2,Tars,Ptpn23,Eif3b,Acsl3,Kif21b,Tbce,Bcs1l,Faah,Nrp1,Prrc2a,Ndufaf7,Rmdn3,Fkbp5,Kcna1,Gsta3,Hars2,Rdx,Crkl,Stx12,Slc25a10,Mtss1l,Hmgcs2,Pfdn5,Ctsa,Dnajc16,Lman1,Msra,Mrps31,Tmem43,Coq9,Rps6ka1,Uckl1,Msto1,Exosc2,Emc7,Ttc3,Rabif,Sec11a,Pik3c2a,Uros,Rps15,Galns,Emc10,Tmed5,Trim21,Cav1,Derl1,Mapkap1,Mrps21l,Enpp2	53
organelle	GO:0043226	Hsp90aa1,Atp2b2,Tars,Ptpn23,Ppfia2,Eif3b,Acsl3,Sorbs2,Aldh9a1,Kif21b,Purb,Tbce,Bcs1l,Faah,Nrp1,Prrc2a,Ndufaf7,Rmdn3,Fkbp5,Kcna1,Map7d2,Shisa6,Gsta3,Hars2,Rdx,Crkl,Stx12,Slc25a10,Mtss1l,Hmgcs2,Ctsa,Lman1,Msra,Mrps31,Tmem43,Coq9,Rps6ka1,Rsu1,Msto1,Exosc2,Emc7,Ttc3,Sec11a,Pik3c2a,Uros,Rps15,Galns,Emc10,Tmed5,Trim21,Rnasel,Cav1,Derl1,Mapkap1,Mrps21l,Dr1,Enpp2	57
intracellular	GO:0005622	Nf1,Hsp90aa1,Atp2b2,Tars,Ptpn23,Eif3b,Acsl3,Sorbs2,Prkag2,Kif21b,Purb,Tbce,Bcs1l,Faah,Nrp1,Prrc2a,Ndufaf7,Rmdn3,Fkbp5,Kcna1,Map7d2,Shisa6,Gsta3,Hars2,Rdx,Crkl,Stx12,Slc25a10,Mtss1l,Hmgcs2,Pfdn5,Ctsa,Lman1,Msra,Mrps31,Tmem43,Coq9,Rps6ka1,Rsu1,Uckl1,Msto1,Exosc2,Emc7,Ttc3,Rabif,Sec11a,Pik3c2a,Uros,Rps15,Galns,Emc10,Tmed5,Trim21,Rnasel,Cav1,Derl1,Mapkap1,Mrps21l,Dr1,Enpp2	60
membrane-bounded organelle	GO:0043227	Hsp90aa1,Atp2b2,Tars,Ptpn23,Ppfia2,Eif3b,Acsl3,Sorbs2,Aldh9a1,Kif21b,Purb,Bcs1l,Faah,Nrp1,Prrc2a,Ndufaf7,Rmdn3,kbp5,Kcna1,Gsta3,Hars2,Rdx,Crkl,Stx12,Slc25a10,Hmgcs2,Ctsa,Lman1,Msra,Mrps31,Tmem43,Coq9,Rps6ka1,Rsu1,Msto1,Exosc2,Emc7,Ttc3,Sec11a,Pik3c2a,Uros,Rps15,Galns,Emc10,Tmed5,Trim21,Rnasel,Cav1,Derl1,Mapkap1,Dr1,Enpp2	52

intracellular organelle	GO:0043229	Hsp90aa1,Atp2b2,Tars,Ptpn23,Eif3b,Acsl3,Sorbs2,Kif21b,Purb,Tbce,Bcs1l,Faah,Nrp1,Prrc2a,Ndufaf7,Rmdn3,Fkbp5,Kcna1,Map7d2,Shisa6,Gsta3,Hars2,Rdx,Crkl,Slc25a10,Mtss1l,Hmgcs2,Ctsa,Lman1,Msra,Mrps31,Tmem43,Coq9,Rps6ka1,Msto1,Exosc2,Emc7,Ttc3,Sec11a,Pik3c2a,Uros,Rps15,Galns,Emc10,Tmed5,Trim21,Rnasel,Cav1,Derl1,Mapkap1,Mrps21l,Dr1,Enpp2	53
endoplasmic reticulum part	GO:0044432	Acsl3,Faah,Fkbp5,Lman1,Tmem43,Emc7,Sec11a,Emc10,Tmed5,Cav1,Derl1	11
bounding membrane of organelle	GO:0098588	Hsp90aa1,Acsl3,Faah,Rmdn3,Fkbp5,Gsta3,Ctsa,Lman1,Msto1,Emc7,Sec11a,Emc10,Cav1,Derl1	14
organelle part	GO:0044422	Hsp90aa1,Ptpn23,Eif3b,Acsl3,Sorbs2,Kif21b,Purb,Faah,Nrp1,Prrc2a,Rmdn3,Fkbp5,Gsta3,Rdx,Crkl,Slc25a10,Mtss1l,Hmgcs2,Ctsa,Lman1,Mrps31,Tmem43,Coq9,Msto1,Exosc2,Emc7,Ttc3,Sec11a,Rps15,Emc10,Tmed5,Trim21,Rnasel,Cav1,Derl1,Mapkap1,Mrps21l	37
organelle membrane	GO:0031090	Hsp90aa1,Acsl3,Faah,Rmdn3,Fkbp5,Gsta3,Slc25a10,Ctsa,Lman1,Tmem43,Coq9,Msto1,Emc7,Sec11a,Emc10,Cav1,Derl1	17
cytosol	GO:0005829	Hsp90aa1,Atp2b2,Tars,Ptpn23,Nrp1,Prrc2a,Kcna1,Gsta3,Crkl,Stx12,Dnajc16,Rps6ka1,Uckl1,Ttc3,Rabif,Uros,Rps15,Cav1,Mapkap1	19
endomembrane system	GO:0012505	Atp2b2,Ptpn23,Acsl3,Faah,Nrp1,Fkbp5,Kcna1,Gsta3,Lman1,Tmem43,Emc7,Sec11a,Emc10,Tmed5,Cav1,Derl1,Mapkap1,Enpp2	18
organelle lumen	GO:0043233	Ptpn23,Eif3b,Sorbs2,Purb,Prrc2a,Fkbp5,Crkl,Slc25a10,Hmgcs2,Ctsa,Mrps31,Tmem43,Exosc2,Ttc3,Rps15,Trim21,Rnasel,Mapkap1	18
mitochondrial part	GO:0044429	Acsl3,Rmdn3,Slc25a10,Hmgcs2,Mrps31,Coq9,Msto1	7
nucleoplasm	GO:0005654	Ptpn23,Eif3b,Sorbs2,Prrc2a,Fkbp5,Crkl,Slc25a10,Ctsa,Ttc3,Rps15,Trim21,Mapkap1	12

Tabel S6 KEGG pathways associated with the DEPs in the As+DIP/As group

Pathway Name	Pathway ID	Genes	Count
Fatty acid degradation	rno00071	Acsl3,Aldh9a1	2
Valine, leucine and isoleucine degradation	rno00280	Aldh9a1,Hmgcs2	2
Protein processing in endoplasmic reticulum	rno04141	Hsp90aa1,Lman1,Derl1	3
Primary bile acid biosynthesis	rno00120	Cyp46a1	1
Glycosaminoglycan degradation	rno00531	Galns	1
Adipocytokine signaling pathway	rno04920	Acsl3,Prkag2	2
Fatty acid biosynthesis	rno00061	Acsl3	1

Table S7 10 important biological process (BP) associated with the reversed DEPs in GO analysis

GO_Name	**GO_ID**	**Genes**	**Count**
catabolic process	GO:0009056	Faah, Enpp2, Cyp46a1, Rdx, Derl1, Ttc3, Exosc2, Gsta3, Hsp90aa1	9
organic substance catabolic process	GO:1901575	Faah, Enpp2, Cyp46a1, Rdx, Derl1, Ttc3, Exosc2	7
ncRNA metabolic process	GO:0034660	Rnasel, Hars2, Exosc2	3
amide biosynthetic process	GO:0043604	Eif1,Hars2,Eif3b	3
nervous system development	GO:0007399	Kcna1, Acsl3, Enpp2, Shisa6, Ttc3, Cav1, Hsp90aa1	7
metabolic process	GO:0008152	Faah,Uckl1,Msto1,Eif1,Rnasel,Acsl3,Enpp2,Cyp46a1,Rdx,Sec11a,Ndufaf7,Psd3,Rps6ka1,Coq9,Nampt,Hars2,Derl1,Ttc3,Eif3b,Exosc2,Dr1,Gsta3,Cav1,Hsp90aa1,Aldh9a1	26
neurogenesis	GO:0022008	Kcna1,Enpp2,Ttc3,Cav1,Hsp90aa1	5
cellular amide metabolic process	GO:0043603	Eif1,Sec11a,Hars2,Eif3b	4
peptide biosynthetic process	GO:0043043	Eif1,Hars2,Eif3b	3
macromolecule metabolic process	GO:0043170	Eif1,Rnasel,Enpp2,Rdx,Sec11a,Ndufaf7,Rps6ka1,Hars2,Derl1,Ttc3,Eif3b,Exosc2,Dr1,Cav1,Hsp90aa1	15

Tabel S8 The pathways associated with the reversed DEPs in KEGG analysis

Pathway Name	Pathway ID	Genes	Count
Primary bile acid biosynthesis	rno00120	Cyp46a1	1
Fatty acid biosynthesis	rno00061	Acsl3	1
RNA transport	rno03013	Eif1,Eif3b	2
Proximal tubule bicarbonate reclamation	rno04964	Slc25a10	1
Protein export	rno03060	Sec11a	1
